# Impact of different aortic valve calcification patterns on the outcome of transcatheter aortic valve implantation: A finite element study

**DOI:** 10.1016/j.jbiomech.2016.03.036

**Published:** 2016-08-16

**Authors:** Francesco Sturla, Mattia Ronzoni, Mattia Vitali, Annalisa Dimasi, Riccardo Vismara, Georgia Preston-Maher, Gaetano Burriesci, Emiliano Votta, Alberto Redaelli

**Affiliations:** a Department of Electronics, Information and Bioengineering, Politecnico di Milano, Via Golgi 39, 20133 Milano, Italy; b UCL Cardiovascular Engineering Laboratory, UCL Mechanical Engineering, University College London, London, UK

**Keywords:** AR, aortic root, AS, aortic stenosis, AV, aortic valve, AVA, aortic valve area, CAS, calcific aortic stenosis, FE, finite element, *R*_C_, commissural radial stent coordinate, *R*_mid_, radial stent coordinate close to leaflets belly, TAVI, transcatheter aortic valve implantation, *ε*_r_, radial strain, *ε*_θ_, circumferential strain, *σ*_I_, maximum principal stress, *σ*_VM_, Von Mises stress, Aortic stenosis, Calcifications, TAVI, Biomechanics, Finite element models

## Abstract

Transcatheter aortic valve implantation (TAVI) can treat symptomatic patients with calcific aortic stenosis. However, the severity and distribution of the calcification of valve leaflets can impair the TAVI efficacy. Here we tackle this issue from a biomechanical standpoint, by finite element simulation of a widely adopted balloon-expandable TAVI in three models representing the aortic root with different scenarios of calcific aortic stenosis. We developed a modeling approach realistically accounting for aortic root pressurization and complex anatomy, detailed calcification patterns, and for the actual stent deployment through balloon-expansion.

Numerical results highlighted the dependency on the specific calcification pattern of the “dog–boning” of the stent. Also, local stent distortions were associated with leaflet calcifications, and led to localized gaps between the TAVI stent and the aortic tissues, with potential implications in terms of paravalvular leakage. High stresses were found on calcium deposits, which may be a risk factor for stroke; their magnitude and the extent of the affected regions substantially increased for the case of an “arc–shaped” calcification, running from commissure to commissure. Moreover, high stresses due to the interaction between the aortic wall and the leaflet calcifications were computed in the annular region, suggesting an increased risk for annular damage.

Our analyses suggest a relation between the alteration of the stresses in the native anatomical components and prosthetic implant with the presence and distribution of relevant calcifications. This alteration is dependent on the patient-specific features of the calcific aortic stenosis and may be a relevant indicator of suboptimal TAVI results.

## 1 Introduction

Transcatheter aortic valve implantation (TAVI) is a minimally invasive procedure currently used for the treatment of aortic stenosis (AS) in symptomatic patients with important contraindications for surgery (Smith et al., 2011, Vahanian et al., 2008). TAVI consists in the percutaneous implantation of a biological heart valve mounted within a metal stent. The latter can be made from Ni–Ti super-elastic alloy, resulting in a self-expandable device, or from elasto-plastic metals (e.g. stainless steel, Co–Cr, etc.) in which case the prosthesis is balloon-expandable. In both cases, the stent expansion pushes the native aortic valve (AV) leaflets against the aortic root (AR). Correct stent expansion is essential to ensure that the device maintains its position after implantation, as well as a correct function of the prosthetic leaflets. TAVI candidates often present calcified aortic leaflets (Stewart et al., 1997) with variable and heterogeneous degrees and patterns of calcium deposits (Thubrikar et al., 1986), which may severely affect the expansion of the stent and, hence, *in vivo* implant outcomes (Detaint et al., 2009, Halevi et al., 2015, Rosenhek et al., 2000, Schievano et al., 2010). Possible complications include dislodgement or migration of the prosthetic device, paravalvular leakage and stroke, potentially associated to the breakdown of calcium deposits (Bax et al., 2014, Ewe et al., 2011, Webb and Wood, 2012). Given the key role of the mechanical interaction between the stent of the prosthetic device, the native AR and, in particular, the native AV, the mechanical analysis of TAVI function within a human AR affected by calcific AS is crucial to quantify and understand the dependency of TAVI outcomes on calcifications severity and patterns.

In this perspective, numerical models represent a powerful tool, due to their inherent capability to analyze the sensitivity of a given system to different factors in a fully controlled and deterministic fashion, while accounting for complex geometry and material mechanical properties. This approach has been increasingly adopted to compute AR biomechanics following TAVI procedures. Anatomically detailed AR finite element (FE) models based on computed tomography have been used to predict the effects of different positioning (Capelli et al., 2012) and of focal calcifications (Wang et al., 2012) on the stent of a balloon-expandable TAVI device within a calcific AV, neglecting the presence of the prosthetic leaflets. More recently, both the implantation of balloon-expandable TAVI devices and the prosthesis diastolic biomechanics have been simulated (Auricchio et al., 2014, Morganti et al., 2014), although through the adoption of potentially relevant simplifying assumptions that may prevent from fully capturing the effects of the calcific disease in terms of stent and prosthetic leaflet distortions. These consisted either in neglecting: i) the presence of the native AV (Auricchio et al., 2014), ii) the presence of the balloon, thus forcing the expansion of the stent through a pre-defined and uniform radial displacement field independent of local increases in the stiffness of the surrounding anatomical structures (Morganti et al., 2014), or iii) the pressure loads acting on the AR wall (Morganti et al., 2014).

Also, different numerical studies have shown that the presence of AV leaflet calcifications can be considered, either through simplified approaches which assume leaflet stiffening (van Loon, 2010, Weinberg et al., 2009) or thickening (Dimasi et al., 2015, Katayama et al., 2013), or adopting more realistic models allowing for the detailed description of calcification locations and morphologies, based on *in vivo* imaging (Halevi et al., 2015, Morganti et al., 2014).

In this paper, we present a numerical study of the implantation of a clinically available and widely used balloon-expandable TAVI prosthesis within an anatomically realistic FE model of the human AR affected by calcific AS. The simulation combines the comprehensive descriptions of all of the steps of the TAVI procedure in a pressurized AR with realistic modeling of different AV calcification patterns, and with the simulation of the prosthesis function throughout the cardiac cycle. The aim of the study is to quantify the effects of different calcification patterns on TAVI outcome in terms of i) stent distortions, ii) prosthetic leaflets diastolic coaptation and systolic opening, iii) stent malposition with associated possible paravalvular leakage, iv) stress concentrations acting on calcifications during TAVI procedure, which may be indicative of increased risk of embolization of calcific material.

## 2 Materials and methods

A three-dimensional AR finite element model was implemented. Three different versions of the model were set, each one being characterized by a different pattern of AV leaflets calcification. For each version, the biomechanics of the AR associated to calcific AS was computed (named CAS simulations), the transapical TAVI procedure with an Edwards SAPIEN® device (Edward Lifesciences Inc.; Irvine, CA) was simulated, and the post-implant biomechanics of the prosthetic valve was estimated throughout a cardiac cycle (TAVI simulations). Simulations were run on the commercial FE explicit solver LS-DYNA© v. 971 (LSTC, Livermore, CA, USA) on an Intel Xeon (2.93 GHz) workstation with 12 processors.

### 2.1 AR geometry

**Fig. 1:**
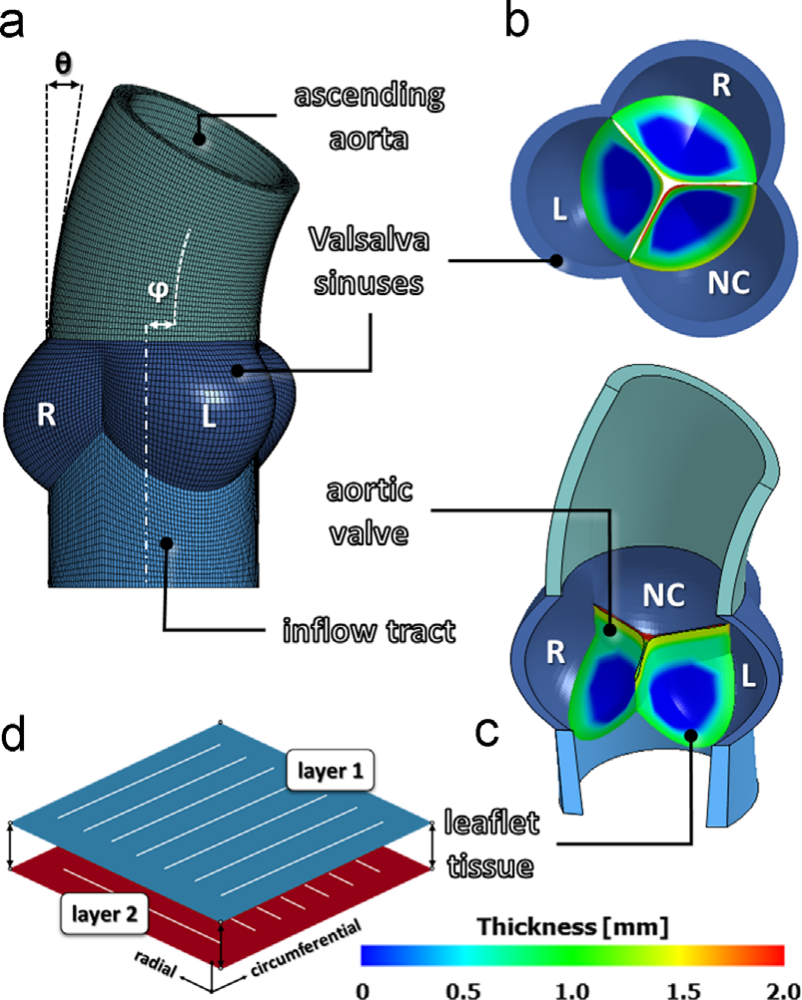
Aortic root geometrical model as reproduced from Sturla et al. (2013): a–c) AR sub-structures, definition of the left (L), right (R) and non-coronary (NC) Valsalva sinuses, and redistribution of the local thickness on each AV leaflet; d) schematic representation of the two-layer FE model adopted to reproduce the macroscopic mechanical response of native AV leaflets, as proposed by Wenk et al. (2012).

The geometry of the AR model was identical to that previously described in Sturla et al., (2013), which accounts for the asymmetry of the three leaflet-sinus units, as well as for the curvature and tilting of the ascending aorta (Fig. 1a and b). The aortic wall, consisting of interleaflet triangles, Valsalva sinuses and ascending aorta, was modeled as a thick walled vessel with a homogeneous thickness of 2.3 mm (Grande et al., 1998), and discretized into linear hexahedral elements with reduced integration (characteristic dimension 0.2–0.4 mm). Dimensions and proportions of the aortic wall were defined averaging *in vivo* measurements from 10 healthy subjects, obtained through cardiac magnetic resonance imaging (Conti et al., 2010). AV leaflets were treated as thin structures and discretized into 4-node shell elements (characteristic dimension 0.2–0.4 mm), with geometry defined based on *ex vivo* data of leaflet surface dimensions and regional thickness variations (Fig. 1c) (Beller et al., 2004, Grande et al., 1998, Kunzelman et al., 1994). *In vivo* and *ex vivo* data were properly scaled to be all consistent with a 24 mm annular diameter (Labrosse et al., 2010).

### 2.2 AR tissues mechanical properties

AR wall tissue was assumed isotropic linear elastic, with a 2 MPa Young modulus and a 0.45 Poisson ratio (Sturla et al., 2013). The mechanical response of AV leaflets was modeled as hyperelastic, anisotropic and incompressible. In the real leaflet tissue, this macroscopic stress–strain behavior is the result of the tissue׳s microstructure, which is characterized by crimped collagen fibers preferentially aligned in the commissure–commissure direction, although with a degree of dispersion. In our model, we reproduced this macroscopic stress-strain behavior through the multilayer approach proposed by Wenk et al. (2012). Across the leaflet thickness, two layers of shell elements with shared nodes were defined (Fig. 1d), which accounted for 55% and 45% of the local leaflet thickness, respectively. The material of the two layers was described as fiber-reinforced, with the fibers oriented along the commissure-commissure and radial direction of the leaflet, respectively, through the invariant-based strain energy function proposed in Quapp and Weiss, (1998) and available in LS-DYNA:

1) $$W=\frac{C_{1}}{2}\left({\bar{I}}_{1}-3\right)+\frac{C_{2}}{2}\left({\bar{I}}_{2}-3\right)+F\left(\lambda \right)+\frac{1}{2}K\ln\left(J\right)$$

where *W* is the energy density function, *Ī*_1_*=tr*(*C*) and *Ī*_2_=½[*tr*(*C*)^2^−*tr*(*C*^2^)] are the first and second invariant of the deviatoric component of the right Cauchy–Green strain tensor *C*, *J=det*(*F*) is the determinant of the deformation gradient *F,* and *K* is the bulk modulus. *F*(*λ*) represents the contribution of fibers along a defined fiber direction and satisfies the following conditions:

2) $$\lambda \frac{\partial F\left(\lambda \right)}{\partial \lambda }=\{\begin{array}{cc} 0 & \lambda <1\\ C_{3}\left[e^{C_{4}\left(\lambda -1\right)}-1\right] & 1<\lambda <\lambda^{*}\\ C_{5}\lambda +C_{6} & \lambda >\lambda^{*} \end{array}$$

where *λ* is the stretch in the fiber direction, *C*_1_–*C*_6_ are material constants, and $\lambda^{*}$ is the stretch value at which collagen fibers are straightened. As in Wenk et al. (2012), *C*_2_ was set to 0, and the linear portion of the function (i.e. for $\lambda >\lambda^{*}$) was neglected by setting $\lambda^{*}$ to 1000. *C*_1_, *C*_3_, *C*_4_ were identified (Table 1) by fitting the homogenized stress on the two layers to experimental data from equi-biaxial testing of leaflet tissue (Billiar and Sacks, 2000). It is worth to stress that this modeling strategy was adopted to effectively capture the stress-strain response of AV leaflets at the tissue level, and not to represent the microstructural architecture of the tissue. A density of 1100 kg/m^3^ was assumed for all the AR tissues (Conti et al., 2010).

### 2.3 AV calcific deposits

**Fig. 2:**
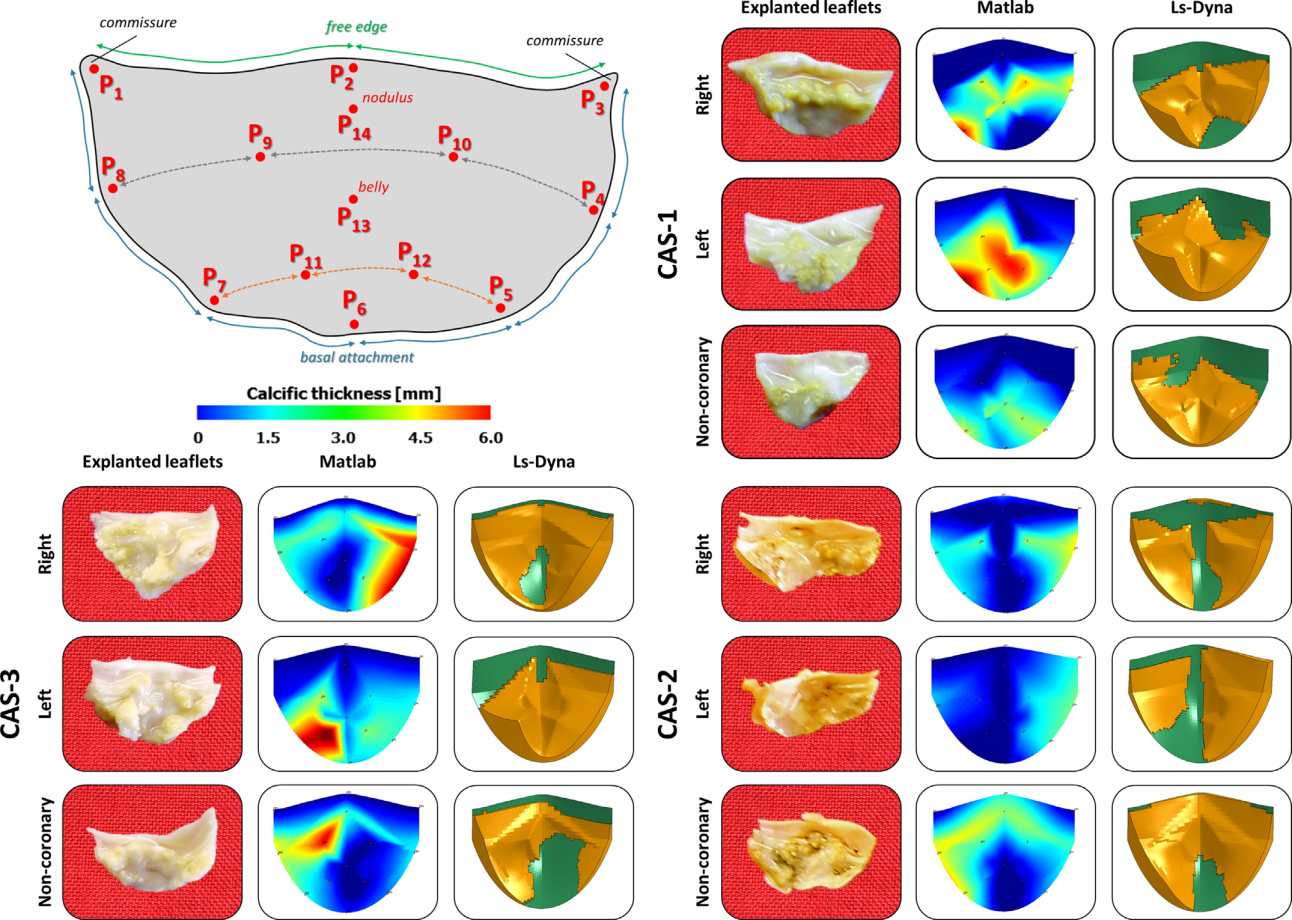
Reproduced numerical stenotic aortic valves CAS-1, CAS-2 and CAS-3, respectively. Experimental *ex vivo* measurements were performed on three explanted human aortic valves on prescribed locations (*P*_1_–*P*_14_); the calcific patterns were then replicated using a dedicated script, and finally integrated in each AR model.

Three AR models with different calcific stenotic AVs (CAS-1, CAS-2 and CAS-3, respectively) were implemented. The corresponding calcification patterns were based on *ex vivo* experimental measurements on three explanted human AVs: local thickness of calcified leaflets was quantified through a digital caliper at 14 selected points of each cusp (Table S1). Through a dedicated Matlab® script (Mathworks Inc., Natick, MA, USA), measures were interpolated to obtain the continuous space-dependent calcific leaflet thickness. The latter was reduced by the space-dependent thickness associated to healthy leaflets (see Section 2.1) to obtain the thickness of calcium deposits only. These were modeled by four layers of hexahedral elements, obtained by extrusion of the underlying shell elements composing AV leaflets (Fig. 2). Based on the histopathological analogy between AV calcific lesions and arterial atherosclerotic plaques (Otto et al., 1994, Wierzbicki and Shetty, 1999), we modeled the stress-strain behavior of calcium deposits as the one of calcific atherosclerotic plaques, which is notably non-linear (Loree et al., 1994, Pericevic et al., 2009). To this aim, we adopted a 1st order Ogden hyperelastic model (Hallquist, 2006):

3) $$W\left(\lambda_{1},\lambda_{2},\lambda_{3}\right)=\sum_{i=1}^{3}\frac{\mu }{\alpha }\left(\lambda_{i}^{\alpha }-1\right)$$

where *μ* and *α* are material constants and were set equal to 13.3 kPa and 24.88 [dimensionless], respectively, based on the least square fitting of the data reported in Loree et al. (1994). A density of 1600 kg/ m^3^ (Loree et al., 1994) and a Poisson ratio equal to 0.495 were defined.

### 2.4 Prosthetic device model

**Fig. 3:**
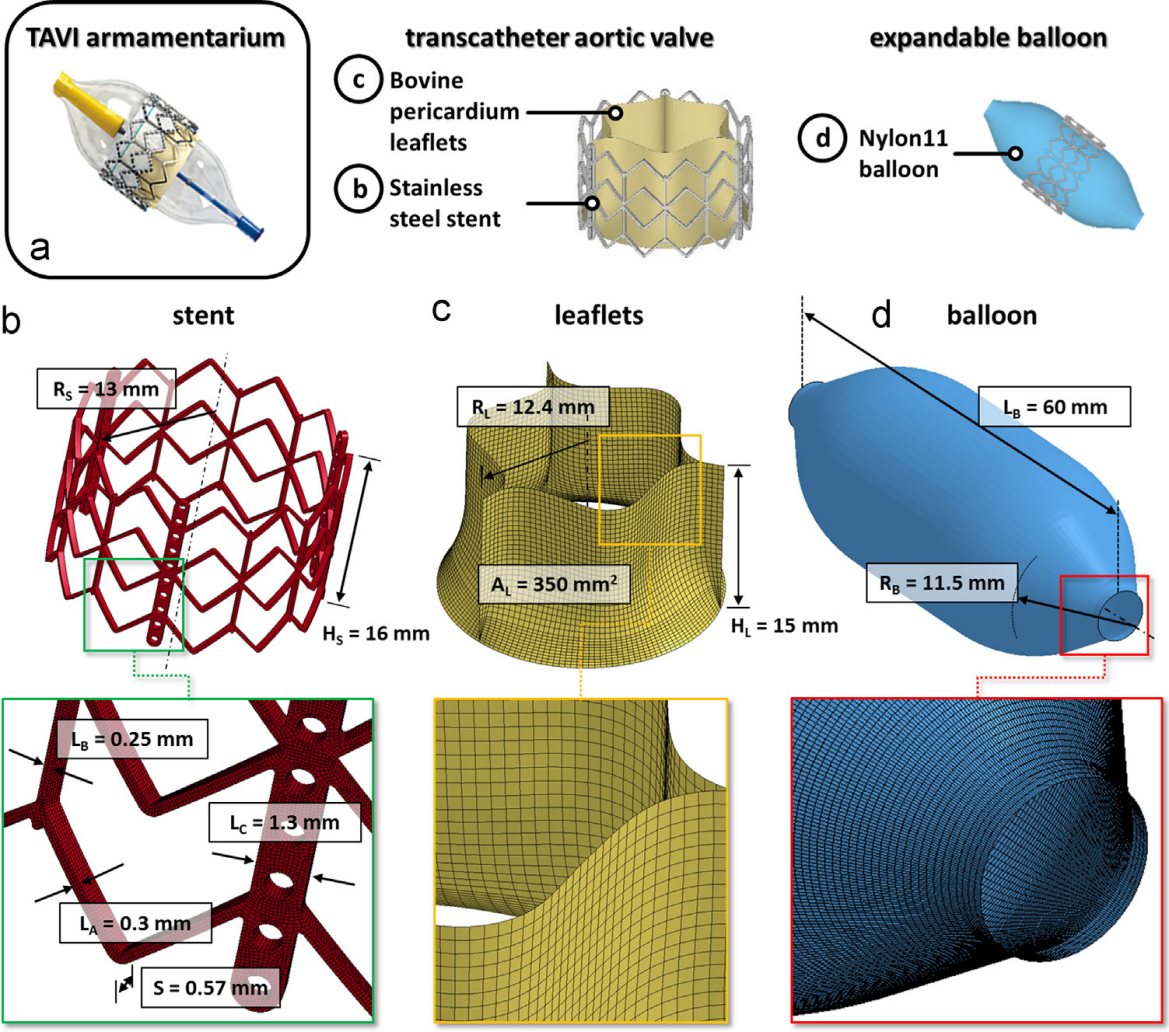
Balloon-expandable transcatheter aortic valve (a) and corresponding FE numerical model (b–d). (b) CAD-model of the TAVI stent: *R*_S_, stent external radius; *H*_S_, stent height; *S*, stent thickness; *L*_A_, *L*_B_, *L*_C_, local stent widths. (c) TAVI leaflets: *R*_L_, leaflet radius; *H*_L_, leaflet height; *A*, leaflet area. (d) Expandable balloon: *L*_B_, balloon length; *R*_B_, balloon radius. For each component a detail of the mesh is proposed in the bottom of the panel.

For the TAVI device, the model of an Edward SAPIEN® size 26 mm (Edward Lifesciences Inc., Irvine, CA, USA) was considered; this size was selected as recommended for implantation into annular diameters ranging between 22–25 mm (Tzamtzis et al., 2013). The geometrical model was based on measurements performed on the real device (Fig. 3a) in the Cardiovascular Engineering Laboratory at University College of London (UCL, London, UK), and consisted of three components:

i. *Stainless*-*steel stent* – The 3D model, depicted in Fig. 3b with its dimensions, was discretized into a uniform mesh of 116928 hexahedral 8-node elements (characteristic dimension 0.1 mm). As in Tzamtzis et al. (2013), the mechanical properties of stainless steel X2CrNiMo-18-15-13 were reproduced by means of a bilinear elasto-plastic model based on a von Mises yielding criterion (*E*=193 GPa, *υ*=0.2, *σ*_Y_(0.2%)=340 MPa and *σ*_U_(48%)=670 MPa); density was set to 8000 kg/m^3^.
ii. *Prosthetic leaflets* – Pericardial leaflets were modeled as equal-sized and with a uniform 0.5 mm thickness (Auricchio et al., 2014), and were discretized into 4-node shell elements (characteristic dimension 0.3–0.5 mm, Fig. 3c). In the real prosthetic device, leaflets are made of glutaraldehyde-treated bovine pericardium. As the fixation process greatly reduces pericardium anisotropy (Langdon et al., 1999), its mechanical behavior can be described as isotropic (Trowbridge et al., 1985). To this aim, we used a 1st order Ogden hyperelastic constitutive model (Eq. (3)), with *μ* and *α* equal to 0.1 kPa and 32.17 [dimensionless], respectively, based on the fitting of data from uniaxial tensile tests performed on 6 prosthetic leaflet specimens obtained from real Edward SAPIEN® prosthetic valves. Tests were performed in distilled water at 37 °C in load-control conditions, using dumbbell-shaped specimen with 16 mm gauge length and 4 mm width. Following 10 preconditioning cycles with 0.5 N peak load, a ramp of load from 0 to 10 N was applied at a displacement rate of 200 mm min^−1^. Density was set to 1100 kg/m^3^, consistently with data from Zioupos et al. (1994).
iii. *Balloon* – The 3D geometrical model of the inflation balloon (Fig. 3d) was discretized into 4-node shell elements (characteristic dimension of 0.25 mm) with uniform thickness of 0.3 mm. Nylon 11 was modeled as a linear, elastic and isotropic material with elastic modulus and Poisson ratio of 0.6 GPa and 0.45, respectively (Tzamtzis et al., 2013), and with a density of 1256 kg/m^3^ (Capelli et al., 2012).

### 2.5 FE simulations

#### 2.5.1 CAS simulations

AR dynamics was simulated as detailed in Sturla et al. (2013). Briefly, after initial pressurization of the aorta to the end-diastolic 80 mmHg pressure, two consecutive cardiac cycles were simulated by applying standard uniform physiological time-dependent aortic (*P*_ao_) and ventricular (*P*_lv_) pressures on the ascending aorta inner wall and on the inner surface of the inflow tract, respectively. The transvalvular pressure (∆*P*=*P*_lv_−*P*_ao_) was applied on ventricular surface of the calcific AV leaflets. The nodes at the proximal and distal end of the aorta were constrained preventing rigid translations. Contact interactions were modeled through the automatic scale-penalty contact algorithm available in LS-DYNA (Hallquist, 2006). Results from the second simulated cardiac cycle were considered for post-processing.

#### 2.5.2 TAVI simulation

**Fig. 4:**
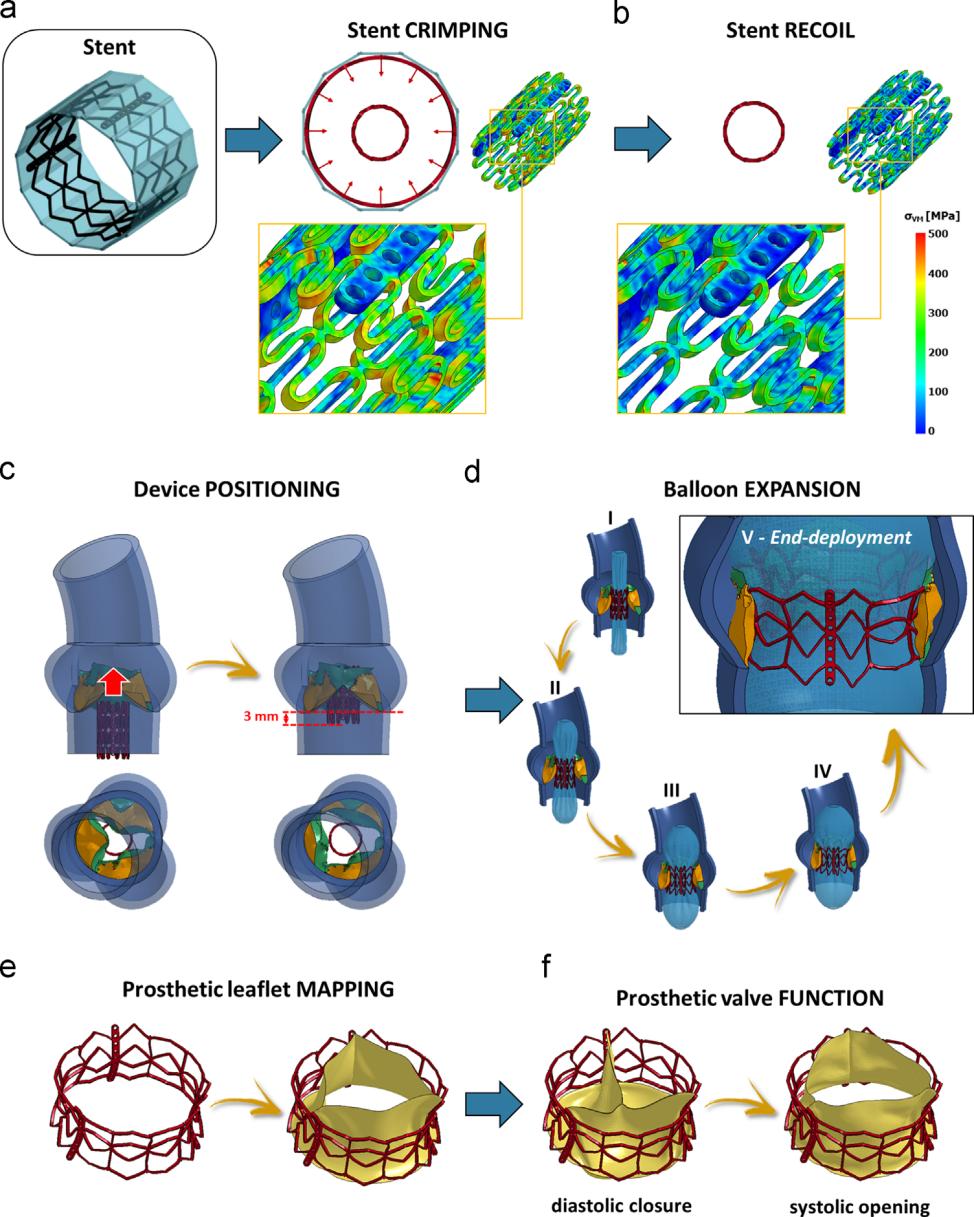
Numerical workflow of the TAVI procedure: initial stent crimping (a) and stent recoil (b); (c) positioning of the device into each CAS-affected aortic root; (d) balloon-driven TAVI deployment and configuration of the TAVI stent at end-deployment (frame V); (e) TAVI prosthetic leaflets mapping onto the deployed stent; (f) assessment of TAVI prosthetic biomechanics during systolic opening and diastolic closure.

The entire TAVI procedure was simulated in each CAS model through 6 steps (Fig. 4):

i. *Stent* -*crimping* (Fig. 4a) – 12 rigid planes, evenly rotated around the stent axis and encompassing the stent, were displaced radially inwards to reduce the stent diameter to approximately 10 mm. With the same procedure, the balloon was crimped separately, reducing its diameter to 9 mm.
ii. *Stent recoil* (Fig. 4b) – The 12 rigid planes were removed so to allow for the radial expansion of the stent associated to its elastic recoil.
iii. *Stent positioning and implantation* – Stent and balloon, with their residual stress and strain fields, were imported in each CAS model pressurized to the systolic configuration with open AV and centered into the AV plane (Fig. 4c), maintaining the lower rim of the prosthesis 3 mm below the AV annulus (Delgado et al., 2010). The balloon within the stent was increasingly pressurized until the stent outer diameter, measured at the stent mid-section, was 26 mm, i.e. slightly larger than the size of the aortic annulus (Al-Lamee et al., 2011) (Fig. 4d).
iv. *Prosthetic leaflet positioning* – As in Auricchio et al., 2014, Dimasi et al., 2015, the prosthetic leaflets were positioned within the stent. Through a set of non-uniform imposed displacements, the nodes lying on the leaflet basal attachment and along the commissures were mapped onto the stent frame and tied to it (Fig. 4e).
v. *TAVI prosthesis function* – Assuming that no further changes could affect the native AR nor the stent, prosthetic leaflets biomechanics was computed throughout two consecutive cardiac cycles by applying to their ventricular surface the time-dependent transvalvular ∆*P* used in CAS simulations (Fig. 4f).

## 3 Results

### 3.1 CAS models

**Fig. 5:**
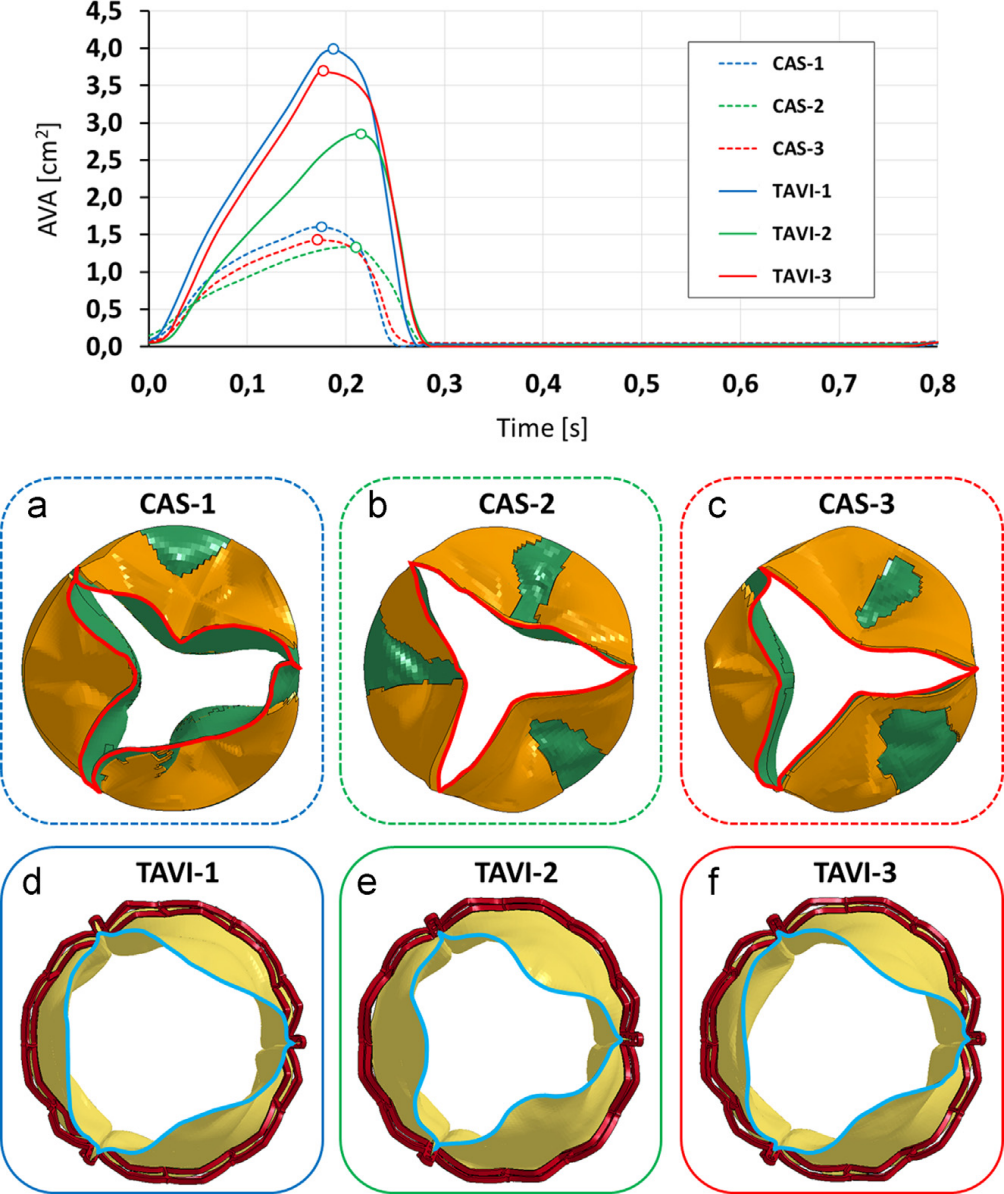
Aortic valve area (AVA) computed throughout a cardiac cycle for the three simulated CAS models and the corresponding TAVI simulations. (a–c) systolic configuration reporting the systolic peak AVA value in CAS-1 (a), CAS-2 (b) and CAS-3 (c) models; (d,e) prosthetic TAVI leaflets at the systolic AVA peak: TAVI-1 (d), TAVI-2 (e) and TAVI-3 (f).

In order to assess whether CAS models correctly replicated AS, the aortic valve area (AVA) vs. time was computed as the extension of the projection of AV free-margin on the annular plane (Fig. 5). During systole, the CAS-affected models presented maximum AVA values equal to 1.60, 1.34, and 1.43 cm^2^ in models CAS-1, CAS-2, and CAS-3, respectively (Fig. 5a–c). These values are slightly above the threshold commonly used to define severe AS (Delgado et al., 2012). At maximum opening, AV orifice was markedly distorted, depending on the AV calcific pattern; asymmetries in the calcification pattern resulted into an asymmetric leaflet profile, and calcifications next to the free margin more heavily limited the leaflet opening motion (Fig. 5b and c).

Also, valvular opening and closing rates were measured by dividing peak AVA by the time needed to reach it from the AV closed configuration and by the time needed to return to complete closure, respectively (Arsenault et al., 1998). In models CAS-1, CAS-2, and CAS-3, the opening rate was equal to 9.1, 6.6 and 7.9 cm^2^/s, respectively, and the closing rate was equal to 21.0, 13.5, and 13.0 cm^2^/s, respectively.

### 3.2 TAVI models

#### 3.2.1 Deployed stent-configuration

**Fig. 6:**
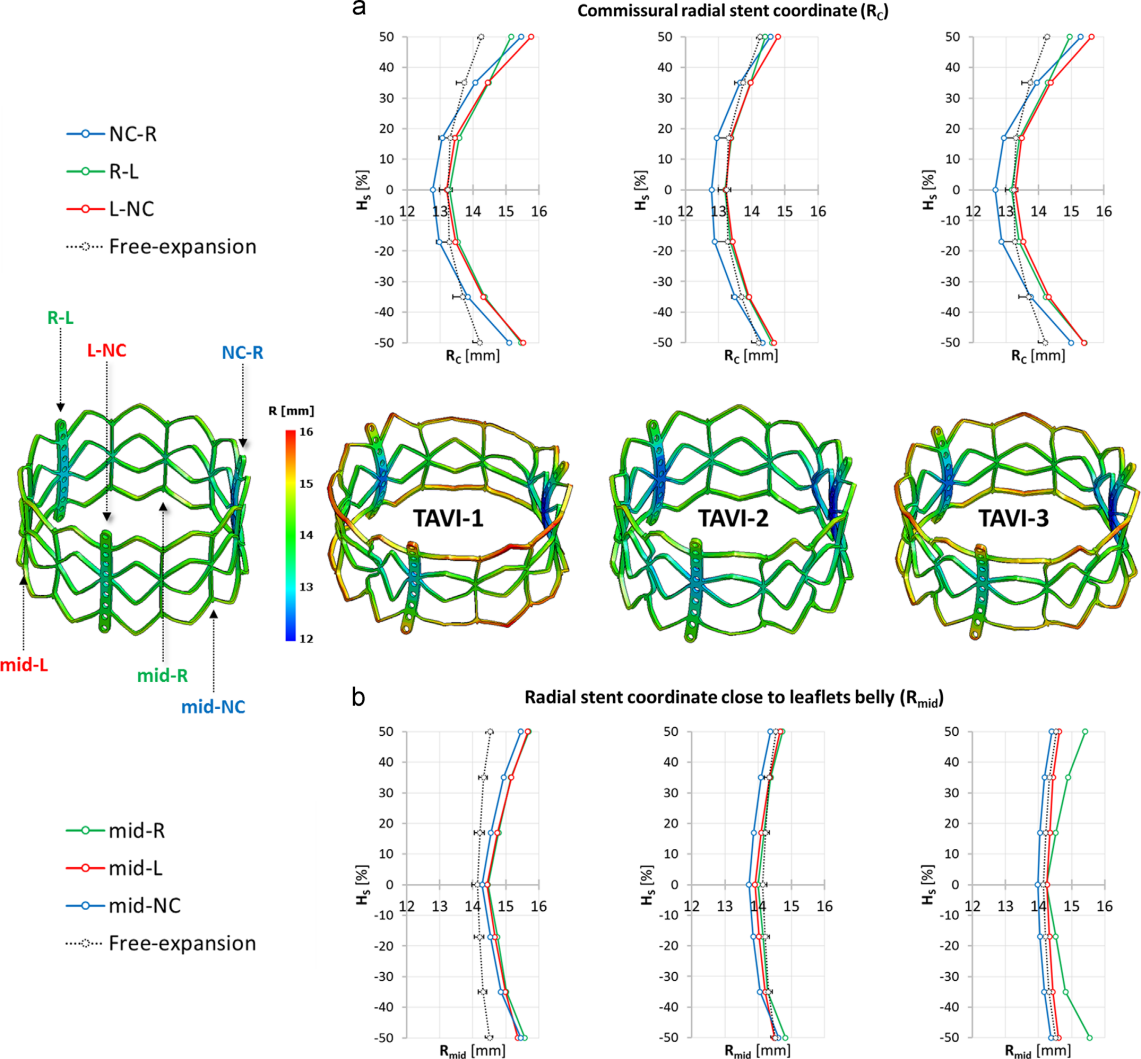
Contour plots of the stent radial coordinate computed after TAVI free-expansion (i.e. in absence of anatomical constraints, on the left) and after TAVI deployment in each simulated CAS model (on the right). The radial stent coordinate was monitored (a) along the commissural struts (*R*_C_; NC-R in blue, *R*-*L* in green and *L*-NC in red) and on (b) the stent sections aligned with the leaflets centerline (*R*_mid_; mid-NC in blue, mid-*R* in green and mid-*L* in red) on seven equally spaced points along the stent axis. For TAVI free-expansion, the mean radial coordinate is reported with dotted-lines, with bars pointing out the minimum and the maximum value of radial coordinate. *H*_S_, stent height. (For interpretation of the references to color in this figure legend, the reader is referred to the web version of this article.)

In all of the three models, following stent deployment within the native AV and balloon removal, the stent was characterized by dog-boning in its longitudinal section, defined as a larger expansion in diameter at the distal portions of the stent, compared with that at the central part of the stent (De Beule et al., 2008). This effect was more pronounced along the commissural struts, where the median stent radius, computed over the three commissures, was 15.15 and 15.10 mm at the distal and proximal end of the device, and 13.20 mm at its mid-section (Fig. 6a). The effect was less pronounced at the stent sections aligned with the leaflets centerline (at about 60° from the commissures), where the median stent radius, calculated over the three leaflets, was 14.8, 14.98, and 14.11 mm at the distal end, at the proximal end, and at the mid-section of the stent, respectively (Fig. 6b). Stent dog-boning was negligible when expanding the stent by balloon inflation without the surrounding anatomical structures (free-expansion). Also, the stent profile depended on the CAS-specific pattern of calcifications: not only it changed from model to model, but also from leaflet to leaflet. The more calcific the leaflet, the more evident the dog-boning effect.

### 3.2.2 Deployed stent stresses

**Fig. 7:**
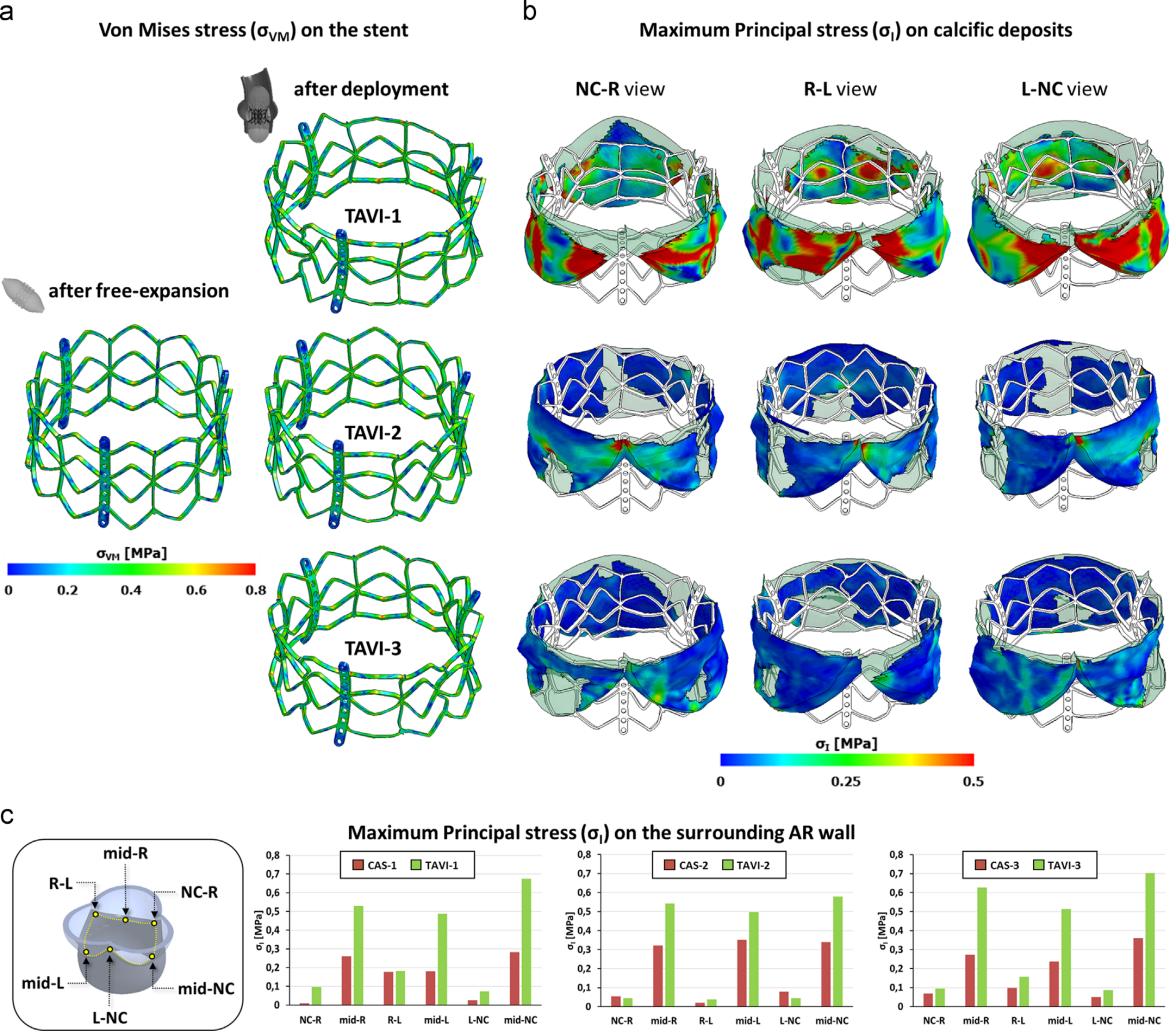
Contour maps of Von Mises stress (*σ*_VM_) on the TAVI stent after free-expansion (a) and after deployment into each simulated calcific AR model (b). Maximum principal stress (*σ*_I_) on the calcific deposits after TAVI deployment in each simulated CAS model: commissural views from NC-R, R-L and L-NC commissure, respectively. NC, non-coronary; R, right; L, left.

For all three models, stent von Mises stresses (*σ*_VM_) following deployment were higher than in the stent expanded without surrounding native structures (Fig. 7a). In particular, in absence of anatomical constraints, peak *σ*_VM_ was 716 MPa and was located at the plastic joints of the stent, while it rose to 812 MPa (+13.4%) in the TAVI-1 model, and 781 MPa (+9.1%) in TAVI-2 and TAVI-3 models.

### 3.2.3 Stent interaction with the surrounding native structures

Native AV leaflets were stretched by the deployed stent, leading to significant stresses on the calcific deposits, whose entity and spatial distribution depended on the calcific pattern. In model TAVI-1, where calcifications run from commissure to commissure affecting the entire circumferential extent of the leaflet, maximum principal stresses (*σ*_I_) were highest (up to 1.5 MPa) and affected a region covering a significant portion of the underlying leaflet. In models TAVI-2 and TAVI-3, where the central portion of the leaflets was partially free from calcifications, peak *σ*_I_ values were lower (up to 0.8 and 0.5 MPa, respectively), and affected only the leaflet commissures (Fig. 7b).

**Fig. 8:**
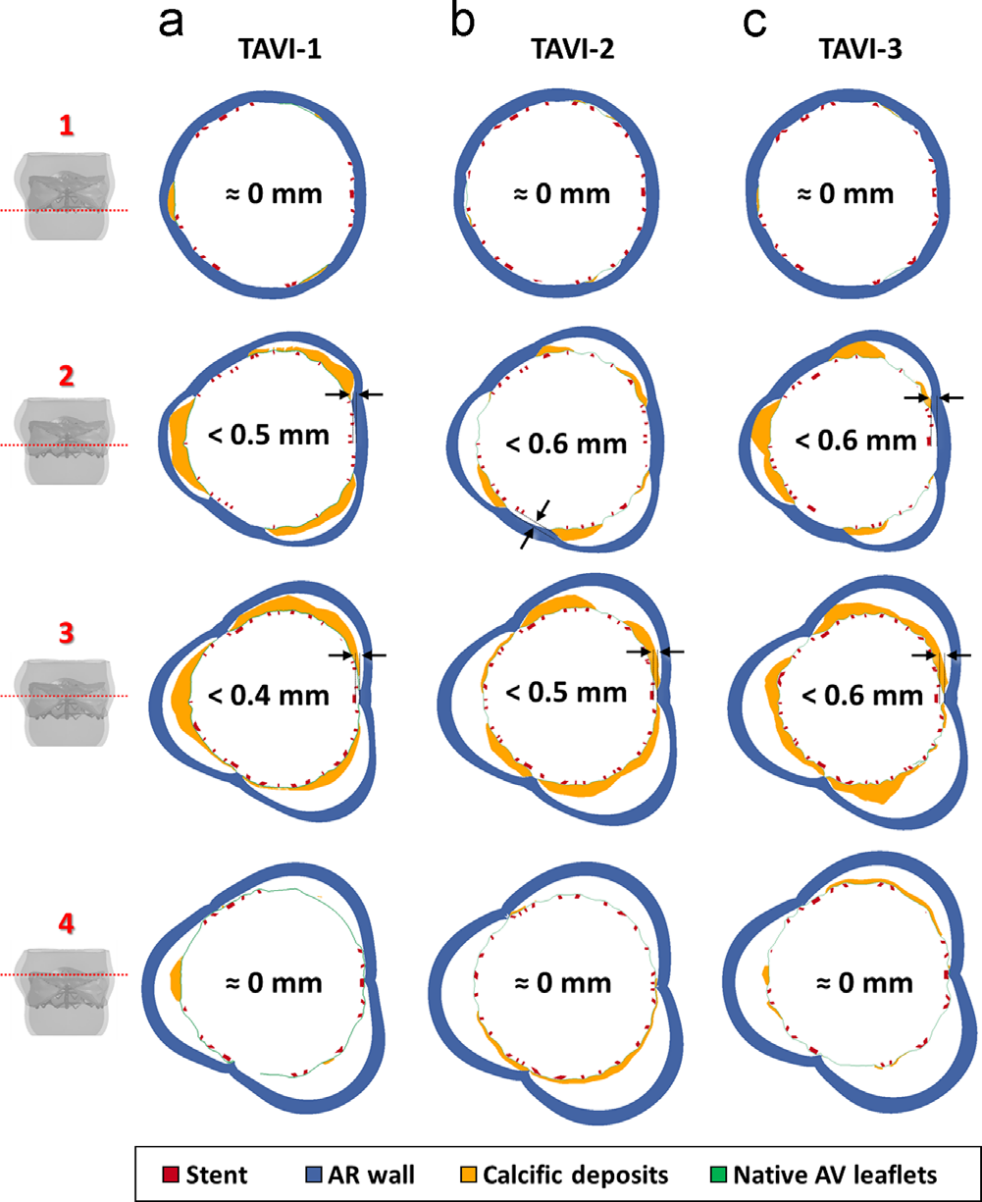
Cross-section view of the gaps, at the end of TAVI deployment, between the stent (red), the calcific AV leaflets (calcific deposits in yellow and native AV leaflets in green) and the AR wall (blue). For each simulated TAVI (a–c), four different cross-sections were considered, equally redistributed between the proximal (cross-section 1) and the distal part (cross-section 4) of the device. (For interpretation of the references to color in this figure legend, the reader is referred to the web version of this article.)

**Fig. 9:**
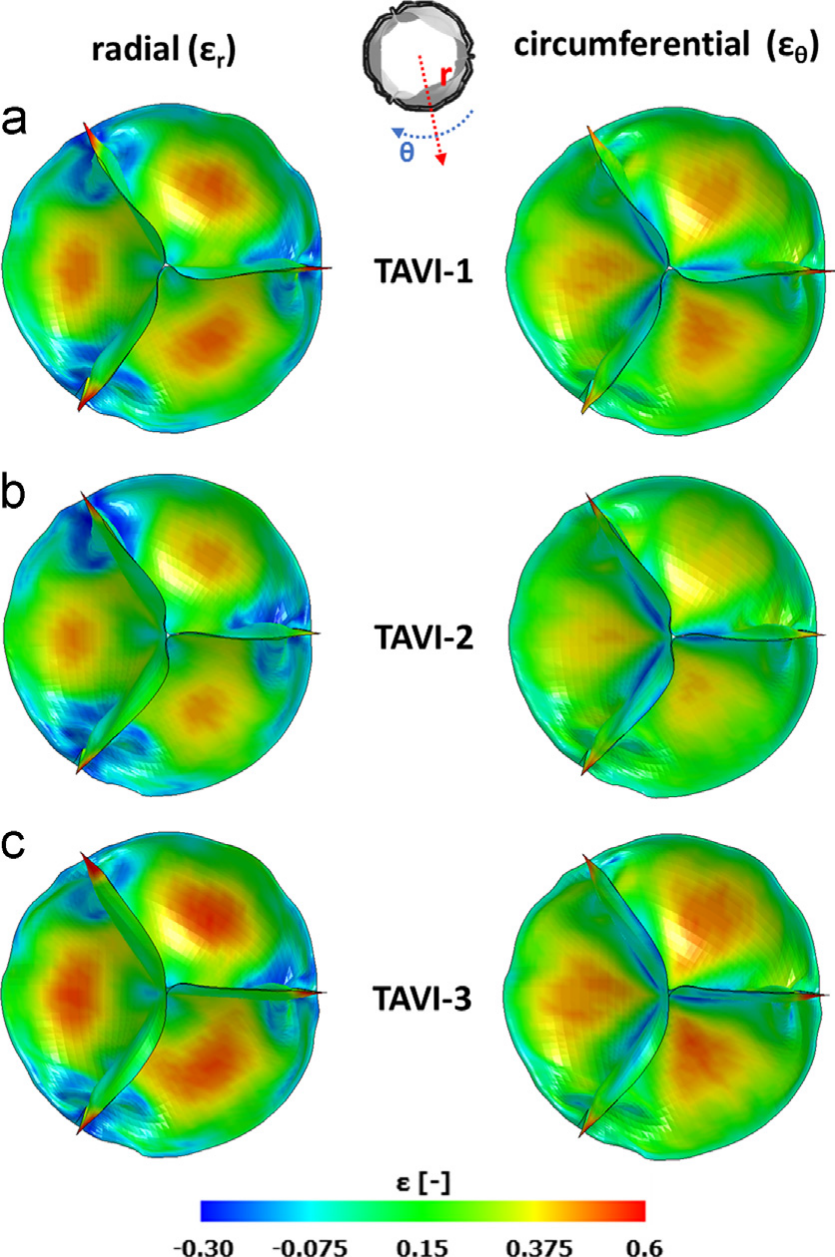
Radial (*ε*_r_) and circumferential (*ε*_θ_) deformation computed on the TAVI prosthetic leaflets, after implantation, during the diastolic phase at the peak of diastolic transvalvular pressure ∆*P*.

The interaction between calcific deposits and the surrounding aortic annulus, determined by the outward relocation of native calcified AV leaflets, affected the stresses acting on the AR inflow tract. Compared to CAS simulations, *σ*_I_ markedly increased. This effect was more evident in the leaflets mid-section and reduced close to AV commissures (Fig. 7c). Also, the interaction between stent, calcific AV leaflets and AV annulus determined local distortions in the stent configuration, assessed by monitoring four cross-sections of the stent equally distributed over the stent axis (Fig. 8). Complete apposition was visible at the two ends of the stent (planes 1 and 4). Sub-millimetric gaps, in the range 0.4÷0.6 mm, appeared along the stent mid-section, between the commissural struts and the AR wall (planes 2 and 3), with stent cross-section resulting more “trilobated” than on the distal and proximal cross-sections (Fig. 9).

### 3.2.4 Prosthetic AV leaflets biomechanics

During systole, implanted prosthetic TAVI leaflets restored a larger AVA compared to CAS simulations, with systolic AVA peak values (Fig. 5d–f) equal to 3.98 cm^2^ in TAVI-1 (+148% vs. CAS-1), 2.86 cm^2^ in TAVI-2 (+134% vs. CAS-2), and 3.68 cm^2^ in TAVI-3 (+152% vs. CAS-3). Accordingly, opening and closing rates substantially increased to 21.1 and 48.2 cm^2^/s in TAVI-1, 13.4 and 38.5 cm^2^/s in TAVI-2, and 20.1 and 36.3 cm^2^/s in TAVI-3, respectively. In all TAVI models, complete diastolic coaptation was obtained (Fig. 5). At peak diastolic ∆*P*, some common features were observed. In particular, leaflets free edge was characterized by pinwheeling, as reported in Young et al. (2011) for the SAPIEN valve with elliptic configuration representative of CAS-related scenarios. The distribution of radial (*ε*_r_) and circumferential (*ε*_θ_) strains was characterized by similar patterns, with high strain (approximately 0.5) regions at the belly and peak strains at the commissures.

## 4 Discussion

Despite its rapid expansion and positive clinical outcome (Webb and Wood, 2012), TAVI still suffers from complications in 5–18% of cases (Delgado et al., 2012). These complications may be related to stent distortions induced by surrounding calcific structures. Though this aspect has been the focus of a number of computational studies (Auricchio et al., 2014, Capelli et al., 2012, Morganti et al., 2014, Wang et al., 2012), the complex mechanical interaction between the native structures and balloon-expandable prosthetic devices has not been analyzed in sufficient detail. This should consider the stent expansion process through a deformable balloon, the presence of native calcified leaflet, the inherently complex and asymmetrical AR anatomy, and the fact that stent deployment is performed within a pressurized AR.

Based on these considerations, we assessed the relation between the presence of relevant AV calcifications and TAVI function through a structural FE model that, to the best of our knowledge, includes for the first time all of abovementioned aspects. In particular, we focused on the potentially different effects of various calcific patterns, by simulating three ARs affected by alternative CAS configurations.

Our results suggested that variations in AV calcific patterns can lead to variations in three aspects of potential clinical relevance.

The first one consists in the implanted stent configuration: local distortions associated with calcifications changed not only with the specific features of the CAS configuration but also within the same model, from AV cusp to AV cusp. Notably, these distortions led to a sub-optimal apposition of the stent on the surrounding native structures. At the mid cross-section of the stent, we estimated sub-millimetric gaps between the stent outer surface and the surrounding anatomy, possibly resulting in paravalvular leakage (Young et al., 2011). The spatial distribution of the gaps over the cross-section was asymmetrical, consistently with the heterogeneous distribution of calcium deposits.

The second relevant aspect consists in the stress distribution produced on calcium deposits. In the model characterized by AV calcifications running continuously from commissure to commissure (CAS-1), stresses were substantially higher than in the other models, and acted on a much larger region. This result is consistent with previous finding, which report higher stiffness for cusps characterized by “arc-shaped” calcifications (Thubrikar et al., 1986). High stresses acting on calcium deposits may be a risk factor for embolization of calcific material and could lead to stroke. This is one of the recurrent complications associated to TAVI on the short- and long-term (Tay et al., 2011).

The third aspect consists in the high stresses acting on the crown-shaped profile of native AV leaflets. These stresses were significant only when simulating TAVI, and affected both the commissural regions and the regions next to leaflets mid-section (located at 60° from the commissures). Of note, the presence of severe calcification in AV and AR has been associated with an increased risk of annular rupture (Delgado et al., 2012).

## 5 Limitations

The main limitations of our modeling approach should be taken into consideration when interpreting results.

First, although realistic in capturing AR asymmetries, the paradigmatic geometrical model we adopted (Sturla et al., 2013) assumes a circular aortic annulus. In CAS patients, the annulus is commonly elliptical, and this geometrical feature may have a great influence on TAVI outcomes, as well as post-operative AV insufficiency (Pontone et al., 2012, Tops et al., 2008). In our study, we obtained a non-circular profile of the stent section, but this was due solely to the presence of AV calcifications. Although this effect is relevant and consistent with clinical evidence (Delgado et al., 2010), it would be further interesting to analyze how it is influenced by an elliptical shape of the annulus.

Second, the mechanical response of aortic wall tissue was described as homogeneous, linear elastic and isotropic, while the real tissue has a hyperelastic and anisotropic stress-strain response, that varies depending on the considered wall region (Choudhury et al., 2009, Gundiah et al., 2008). In principle, this assumption could impact on the mechanical interaction between the aortic wall and the stent, and thus on the stent deployed configuration. However, aortic wall non-linearity and anisotropy are not as marked as in AV leaflets (Choudhury et al., 2009, Gundiah et al., 2008); as a result, the inaccuracies associated to our simplifying assumption are reasonably limited, at least on the deployed configuration of the stent, as suggested by previous studies (Russ et al., 2013).

Third, stent positioning and deployment was simulated for an optimal implant condition. The potential risks highlighted by our numerical analyses are likely to be further exacerbated in the case of malposition of the prosthetic valve.

Fourth, we computed the presence of gaps between the stent and the surrounding anatomical structures. However, in order to assess the impact of these gaps in terms of paravalvular leakage, a more complex fluid–structure interaction (FSI) approach should be adopted.

Fifth, we only simulated three different patterns of AV calcification. In order to reflect the wide spectrum of calcific patterns commonly observed in clinical cases, a much larger number of models should be analyzed.

Sixth, and most relevant, it was not possible to validate our models through the quantitative or semi-quantitative comparison vs. an experimental equivalent of the modeled AR. This limitation affects most of the state-of-the-art numerical studies on TAVI procedures (Auricchio et al., 2014, Cosentino et al., 2015, Morganti et al., 2014); yet, it calls for a cautious interpretation of our results, even though these appear reasonable, in light of the assumptions we made. In particular, the assumptions on native tissues mechanical response inherently affect computed local strains and stresses. However, the presented comparison of the different CAS and TAVI simulations highlights clear features which provide important indications on the biomechanical interaction between balloon-expandable transcatheter valve and calcified host anatomies (Bosi et al., 2015), and confirm the potential role of numerical modeling as a tool for the comparative analysis of different scenarios.

## Conflict of interest

All authors declare no conflict of interest.

## Funding sources

The work was partially funded by the Welcome Trust (095747/Z/11/Z) and the British Heart Foundation (PG/13/78/30400).

## Figures and Tables

**Table 1:** Coefficients of the constitutive model proposed by Quapp and Weiss (1998) identified based on ex vivo data from equibiaxial testing of porcine AV leaflets (Billiar and Sacks, 2000).

	*α* [deg]	*C*_1_ [kPa]	*C*_2_ [kPa]	*C*_3_ [kPa]	*C*_4_ [dimensionless]
**Layer 1** (commissure–commissure direction)	0	21.598	0.0	0.01	32.93
**Layer 2** (radial direction)	90	21.598	0.0	0.00075	35.55

## Footnotes

Appendix A Supplementary data associated with this article can be found in the online version at http://dx.doi.org/10.1016/j.jbiomech.2016.03.036.
