# Supplementary material for: Impact of different aortic valve calcification patterns on the outcome of transcatheter aortic valve implantation: A finite element study
Source: J Biomech. 2016 Aug 16;49(12):2520–30. doi: 10.1016/j.jbiomech.2016.03.036 (PMC5038160; doi:10.1016/j.jbiomech.2016.03.036)
Supplement: Supplementary file 1 — Supplementary material [file mmc1.docx]

**SUPPLEMENTARY MATERIAL**

**Table S1**

Local values of thickness of the calcific deposits on each AV cusps. Values are reported in mm.

|  | CAS-1  C_1_ [KPa]  C_2_ [KPa] | | | CAS-2  C_4_ [-] | | | CAS-3 | | |
| --- | --- | --- | --- | --- | --- | --- | --- | --- | --- |
|  | L | R | NC | L | R | NC | L | R | NC |
| P_1_ | 0.0 | 0.0 | 0.0 | 0.0 | 0.0 | 0.0 | 0.0 | 0.0 | 0.0 |
| P_2_ | 0.0 | 0.0 | 0.0 | 0.0 | 1.3 | 2.4 | 0.0 | 0.0 | 0.0 |
| P_3_ | 0.0 | 0.0 | 0.0 | 2.4 | 0.0 | 0.0 | 0.0 | 0.0 | 0.0 |
| P_4_ | 1.3 | 3.4 | 0.0 | 2.9 | 3.7 | 2.4 | 2.3 | 5.5 | 2.2 |
| P_5_ | 2.5 | 0.0 | 3.4 | 2.6 | 2.4 | 1.5 | 2.5 | 5.5 | 1.4 |
| P_6_ | 2.6 | 0.0 | 1.6 | 0.0 | 0.0 | 0.0 | 2.0 | 1.6 | 0.9 |
| P_7_ | 5.9 | 5.3 | 3.4 | 0.0 | 2.2 | 3.7 | 5.6 | 2.1 | 2.4 |
| P_8_ | 2.0 | 0.0 | 1.4 | 1.7 | 2.4 | 3.7 | 1.3 | 2.7 | 3.5 |
| P_9_ | 3.8 | 3.6 | 0.0 | 0.9 | 2.8 | 2.7 | 3.3 | 2.2 | 5.3 |
| P_10_ | 0.0 | 4.1 | 1.8 | 2.0 | 3.1 | 2.3 | 2.1 | 1.8 | 0.0 |
| P_11_ | 3.6 | 2.1 | 1.9 | 0.0 | 1.0 | 1.4 | 6.3 | 0.0 | 1.3 |
| P_12_ | 4.7 | 0.0 | 3.2 | 1.8 | 0.0 | 0.0 | 3.0 | 2.8 | 0.0 |
| P_13_ | 4.9 | 3.2 | 2.8 | 0.0 | 0.0 | 0.0 | 1.8 | 0.0 | 0.0 |
| P_14_ | 1.5 | 0.0 | 0.0 | 0.0 | 0.0 | 3.0 | 0.0 | 2.0 | 2.5 |
